# General practitioners and management control through guidelines: a qualitative study of its effects on their practice

**DOI:** 10.1186/s12875-025-03171-8

**Published:** 2026-01-10

**Authors:** Jens Lundegård, Åsa Grauman, Niklas Juth, Linus Johnsson

**Affiliations:** 1 Centre for Research Ethics & Bioethics, Department of Public Health and Caring Sciences, Uppsala University, Box 564, 751 22 Uppsala, Sweden; 2 Centre for Clinical Research Sörmland, Uppsala University, Eskilstuna, Sweden

**Keywords:** Clinical decision-making, Clinical practice guidelines, Care pathways, General practitioners, General practice, Qualitative research, Patient-centred care, Healthcare governance, Management control

## Abstract

**Background:**

Changes in healthcare governance and the rise of evidence-based medicine (EBM) have over the last decades contributed to an increase in guideline-driven management of general practice. There is a lack of recent studies investigating how this continuous development affects the practice of Swedish general practitioners (GPs) from a broad perspective. Thus, this study aims to explore how Swedish GPs relate to management control through guidelines, how it affects their daily practice and work environment, and how they reflect on its consequences.

**Methods:**

We conducted semi-structured, face-to-face interviews during 2024 with 11 GPs from all across Sweden. The transcribed interview data were analysed using thematic analysis.

**Results:**

We constructed three themes, each representing a field of tension created by guidelines: (1) Torn between high ambitions and their resulting negative side effects, (2) Guidelines promote measurable over unmeasurable knowledge, and (3) Although autonomy in relation to guidelines is highly valued, there are compelling reasons to submit.

The first theme reflects a broad agreement on the benefits of guidelines and support of the growing ambitions they reflect. However, guidelines also result in increasing work-load and reduce flexibility in healthcare collaboration. The second theme highlights that because guidelines tend to prioritise measurable over non-measurable knowledge, other aspects of GPs’ professional skills risk being underused and underdeveloped. The third theme captures how GPs exercise a high degree of autonomy in relation to guidelines, yet occasionally relinquish their clinical discretion. These tensions may result in side-effects such as a deteriorating work environment, crowding-out effects, fragmented healthcare, and potentially reduced quality in areas of general practice that are difficult to measure.

**Conclusions:**

While management control through guidelines entails many benefits, the participants in this study also reported several adverse effects on both the quality of care and the work environment. Promoting quality by organizing healthcare through increasingly complex guidelines may seem like a natural approach in a system that focuses strongly on measuring outcomes, but it is also important for healthcare decisionmakers and guideline developers to acknowledge its potential side effects.

**Supplementary Information:**

The online version contains supplementary material available at 10.1186/s12875-025-03171-8.

## Background

Over the last decades, the conditions for general practitioners (GPs) in Western countries to independently manage their work have changed, driven by shifts in healthcare governance and management [1–4]. A number of contemporary societal phenomena are considered to have contributed to this development [5–7]. The first is the expansion of bureaucratic governance, which, in combination with the emergence of an 'audit society', places a greater focus on measures such as monitoring, standardization, accountability, performance measurement, evaluations, and audits within organizations [6–9]. Secondly, there is an increasing trend towards market-based governance including competitive contracting, patient-choice, and use of financial incentives [5–7]. A third phenomenon is the emergence of evidence-based medicine (EBM) from the early 1990 s onward—a paradigmatic shift that has influenced healthcare professionals’ decision-making by emphasizing the use of the best available evidence from medical research [10]. In the wake of EBM, clinical practice guidelines have proliferated, with the stated goal of enhancing quality, increasing effectiveness and safety, and reducing practice variation [11, 12].

However, guidelines directed at GPs are often more than just tools for providing recommendations for good practice based on the best available evidence. In addition, they assist in the management of healthcare by serving as rationing tools for cost containment and by providing standards for audits and the assessment of healthcare personnel [11, 13–17]. Although there is no official terminology, several concepts are consistently used to describe different forms of guidelines. For instance, while ‘clinical practice guidelines’ typically help clinicians to make decisions in specific situations, ‘protocols’ and ‘care pathways’ are more comprehensive, outlining clearly defined sequences of steps that should follow a clinical decision [11, 13, 17, 18].

How management control affects the medical profession has been the subject of extensive research and theorizing across different research traditions, focusing on the effects on the sociocultural status of the profession, particularly in relation to privilege, power, and social behaviour [3, 19–21]. The direct consequence of management control through guidelines on the clinical practice of GPs have been less studied [20–23].

Regarding clinical autonomy, existing qualitative research shows that GPs sometimes struggle to maintain their professional integrity in relation to clinical guidelines [15, 20]. Survey data show that doctors have experienced a loss of control over both work organization and working conditions since the 1990 s [24]. Meanwhile, other research also reveals that, in terms of clinical decision-making, GPs often deviate from recommendations, and that nonadherence to prescription guidelines is prevalent [15, 23, 25, 26].

Another area often covered in research investigating GPs’ experiences with clinical guidelines is the issue of trustworthiness and usability. This research indicates that GPs generally trust that clinical guidelines can enhance patient safety and help them stay up to date with the latest medical knowledge [15, 22]. However, uncertainty about both the motives behind the guidelines and their usability in the complex reality of general practice seems to be prevalent [15, 16, 22, 23, 27]. More specifically, findings suggest a perceived increase in the risk of overinvestigation, overtreatment, and polypharmacy, especially among elderly or multimorbid patients [22]. Additionally, there is a concern that guidelines can jeopardize doctor—patient communication and relationships and hinder the GP’s ability to show empathy [16, 23, 28].

Clinical guidelines are also reported to make the work easier by providing practical support in the clinical encounter [21, 27], and to alleviate some of the burden of decision-making responsibility from the GP [15, 23, 27]. However, findings also suggest that guidelines contribute to an increased workload for GPs and may lead to a fear of being subjected to professional review [16, 22, 25, 29, 30].

Earlier studies have investigated GPs’ experiences of guidelines from a narrow perspective, such as barriers to their use, and it has been unusual to allow GPs themselves to define what should be included in the concept of guidelines [23, 27]. Furthermore, there are few recent studies, and none have explored this issue from a broad perspective within the Swedish context—one characterized by GPs being salaried employees and primary care being provided in larger practices compared to most other countries [31–34].

The aim of this study is to provide up-to-date knowledge on how contemporary management control through guidelines may affect the clinical work of Swedish GPs. We will more specifically explore how GPs relate to the management control, how it affects their daily practice and work environment, and how they reflect on its consequences.

## Methods

### Design

We chose a qualitative design based on semi-structured interviews, aiming to gather narratives from lived experience in order to obtain detailed, rich descriptions of the research subject.

### Reflexivity

To enhance reflexivity, and thereby trustworthiness, we actively reflected throughout the study on our own preconceptions and underlying opinions, acknowledging their contribution as a valuable resource when shaping the research. For instance, sharing the same profession as the study participants (as in the case of JL and LJ), and thus being deeply familiar with the subject and the working conditions of the profession, can be advantageous when conducting and interpreting the interviews. However, we have also been aware that such familiarity may influence the interviews in unanticipated ways, may affect the interpretation, and hinder the ability to recognize new patterns in the data. Meanwhile, half of the research team (NJ and ÅG) are not medical doctors and therefore contributed a different perspective throughout the research process, from planning to analysis.

### Participants and setting

To get perspectives from doctors with extensive experience from work in primary health care we chose to only recruit specialists in general practice. In Sweden, this means working at least 5 years as a resident physician before qualifying as a specialist. We primarily used convenience sampling and snowballing, coupled with some strategic sampling, to ensure a diverse group in terms of geographic location, age and sex. Furthermore, a few GPs were recruited deliberately based on their experience of guideline development to ensure the inclusion of that perspective.

All participants were contacted and asked for participation by email, including information about the researchers and the purpose of the study. Roughly half of the invited participants did not respond to the invitation email or chose not to participate for unknown reasons.

In total 11 GPs participated in the study, six women and five men, aged between 39 and 65 years (median age 51). Their experience as specialists in general practice ranged from 4 to 27 years (median 9 years). They worked in different regions from Southern to Northern Sweden, in metropolitan and rural areas, and in publicly as well as privately run primary care centres. None of the participants had a personal relation with the interviewer prior to the study.

### Data collection

The interviews were conducted between January and August 2024. All interviews were conducted face-to-face with only the participant and researcher present. The participants were free to choose a location of their preference. Thus, some interviews were conducted at the participant’s workplace, others in secluded places in public, and some in the participant’s home. The interviews were semi-structured and based on an interview guide (Supplement 1) that was continuously adapted throughout the study to obtain a richer material. When developing the interview guide we were careful to ensure balance by including questions about positive as well as negative experiences of using guidelines. During the interviews we deliberately refrained from defining ‘guidelines’ to avoid the risk of overlooking previously unconsidered perspectives. We also made sure to ask about both positive and negative aspects of using guidelines.

The interview questions revolved around the following themes:

- The GPs’ perception of their own autonomy in relation to guidelines
- Guidelines and the quality of healthcare provided
- The doctor-patient relationship and how it may be affected by guidelines
- The effects of guidelines on the work environment
- Strategies and potential use of guidelines when encountering challenging patients, with ‘challenging’ being defined by the GP

To help the interviewed GPs begin illustrating their opinions with concrete cases from their practice, most interviews began by asking them to describe a patient encounter that they had perceived as challenging. Follow-up questions explored what made the situation challenging, how the GP handled it, and the potential use of guidelines in that context.

All interviews were audio-recorded and transcribed verbatim. The duration of the interviews ranged from 49 to 85 min (mean 61 min). After 11 interviews, information power [35] was deemed sufficient, despite the broad study aim. This judgement was based on the wealth of information in the interviews, resulting from the highly specific sample of study participants relevant to the study, along with the high-quality dialogue.

### Data analysis

The interview data were analysed using thematic analysis [36]. The process began by familiarizing ourselves with the dataset by reading the entire text. We then identified data extracts of interest, and labelled them with one or more codes. In this phase, we aimed to code as many potential patterns as possible, to ensure nothing of interest was left out. JL did most of the coding and ÅG also independently coded part of the text to enhance discussions about interpretations. After the initial coding, the next step involved sorting the codes in search of themes and sub-themes relevant to the study’s aim and research questions. The themes were then reflected upon, reorganized, and renamed several times before we arrived at the final version. During the analysis process, the whole research team openly discussed their interpretations and actively contributed by reviewing the suggested codes and themes in relation to the text to enhance reflexivity.

## Results

During the thematic analysis, three different themes relating to the aim of the study were constructed (Table 1). Each theme is related to a specific field of tension that was identified in relation to the use of guidelines. The themes were further divided into sub-themes for increased clarity. The descriptions of the themes are illustrated with quotations from the participants, including a number representing the participant.

**Table 1:** Overview of the themes and sub-themes

1: Torn between high ambitions and their resulting negative side effects - Guidelines improve health, efficiency and equality - Guidelines cause work proliferation - Ambitions to rationalise the primary-secondary care interface hinder collaboration
2: Guidelines promote measurable over unmeasurable knowledge - Algorithmic thinking may suppress other forms of knowledge - Guidelines may impair clinical judgement - Guidelines are ill-suited for some patient categories
3: Although autonomy in relation to guidelines is highly valued, there are compelling reasons to submit - Exercising a high degree of clinical autonomy - Coping with high demands through adaptation - Experience, fear of making mistakes and local workplace-culture influence autonomy

### 1. Torn between high ambitions and their resulting negative side effects

The first theme concerns the tension between, on one hand, the growing ambitions in healthcare expressed through increasingly complex guidelines, and on the other hand, the sense that this development is progressing without consideration for, or understanding of, the overarching negative effects it may cause.

#### Guidelines improve health, efficiency and equality

A recurring theme was the strong conviction that evidence-based medicine and clinical guidelines improve health outcomes and are crucial for work in contemporary health care. Clinical guidelines help quickly access the most up-to-date information on medical conditions, which would be impossible to obtain through other means, and provide support during clinical encounters. They also contribute to faster spread of new medical knowledge for use in clinical practice. Thus, evidence-based practice evokes positive emotions, such as pride, and contributes to work satisfaction.

*When treating a patient with asthma, you also follow guidelines. That way, you save lives.*

– *GP #1*

There was also a belief that guidelines help protect patients from the GP’s own shortcomings, as well as other incompetent doctors and bad practice. That is also why some of the GPs considered it wrong to act without the support of guidelines.

*Patients coming here should not suffer because of my own shortcomings or a bad day, they should receive equal care. It’s hard to admit, but you’re blind to your own shortcomings. […] That’s why guidelines exist—as a safety net, if you follow them.* – GP *#*4

Several of the GPs spoke positively about the aspect of management control that involves audits and local comparisons between doctors, in areas such as lab testing, radiology, and treatments. Such comparisons commonly use guideline recommendations as the benchmark. This improves the quality of care and gives GPs an opportunity to review their own practice in collaboration with colleagues, thereby enhancing decision-making confidence. The guidelines also directly impact the GP’s decision-making confidence and sense of security by providing clear instructions for concrete situations, leading to faster decision-making and improved work efficiency.

*I don’t believe it’s the guidelines that give me trouble, quite the opposite. They make you read up on the subject, which makes you faster.* – GP *#*1

In addition to improving medical quality for patients, there was also a belief that management control enhances equality and fairness in healthcare across the country, which is seen as an independent positive aspect when judging quality of healthcare.

*National guidelines are a positive thing. Aiming to ensure equal and high-quality healthcare for everyone.* – GP *#*3

#### Guidelines cause work proliferation

The GPs frequently mentioned how new measures are continuously added to their existing work with the good intention of improving healthcare quality. New or updated guidelines often introduce additional measures to implement without removing others to counterbalance the overall workload. Examples include lowering the bar for more advanced investigations, increasingly strict treatment targets, medical safety-netting such as annual lab check-ups when prescribing certain drugs, increased demands for detailed charting, and more requirements for documentation in various registries. While each task may be small on its own, it is the huge cumulative effect of these tasks that makes it difficult to maintain a sustainable pace of work. Another time-consuming task is ensuring compliance with potential audits by documenting extra carefully in the medical record. This additional documentation seemed prevalent, even though most GPs did not appear to be overly concerned about the possibility of being audited.

*You document to keep track of what you’ve done and to help the patient. But we also document quite a lot to prove what we have done. That’s the trend in today’s society - you are not allowed to make mistakes.* – GP *#*5

The GPs provided numerous examples on how guideline development is increasingly leading to stricter and more detailed criteria for when patients can be referred to secondary specialized healthcare. Whereas it was previously possible to refer the patient when the GP deemed necessary, it is now more common for the referral to be rejected with reference to details in formal care agreements—guidelines that specify the criteria a referral must meet to be accepted. These kinds of guidelines were seen more as a rationing tool rather than being focused on the patient’s best interest.

*Previously, you could send referrals for different investigations, and someone would review and prioritise them based on the referral. Now I wonder, do the recipients have any medical knowledge? They return referrals due to lack of information about BMI in the referral, even when I describe severe sciatic symptoms with pain and motor loss.*

– GP *#*8

The proliferation of tasks, increasingly detailed referral requirements, and additional documentation required for potential audits all lead to a growing workload for the GPs—contributing to a deteriorating work environment.

*The administrative work of chasing target levels and similar tasks leads to more work, frustration, crowding-out effects and stress. We can’t cope with it.* – GP *#*4

The increased workload was also perceived as a risk to healthcare quality provided by GPs due to crowding-out effects. When the GPs have to work harder with less time for every task, the result is a sense that there is sometimes insufficient time to make well-founded decisions during individual patient encounter, or to properly prioritise between different patients.

*The public expects us to provide this basic health care, but we can’t right now. Should I then strive for decimals precision in LDL levels or check creatinine levels for everyone? I understand that this can have a significant effect on individual patients, but what are the crowding-out effects?* – GP *#*4

The GPs further discussed how increased management control through guidelines may negatively impact the entire healthcare system by raising costs and causing unintentional crowding-out effects.

*For every diagnosis there is always more you can do—more for diabetes, more for osteoporosis, more for wound treatment, and more for everything. But the resources won’t be enough to do more of everything.* – GP *#*7

#### Ambitions to rationalise the primary-secondary care interface hinder collaboration

Some GPs described how the current system, with care agreements that strictly define the responsibilities of each healthcare setting in an ambition to rationalise healthcare, leads to less flexibility and a worsened collaboration between doctors. In the short term it may be regarded positively when the care agreements are designed to increase power for doctors in one healthcare setting at the expense of others. This was illustrated by several GPs mentioning that they approve of guidelines that make it impossible for secondary care doctors to reject certain referrals. However, in the long run, this reinforces a mindset where everyone focuses only on their own role instead of sharing responsibility for the patient and helping each other.

*In this guideline-developing committee where I participated, people have stood up, shouting at each other. Harsh words are exchanged– ‘This is your job’, and ‘We can’t perform genetical investigations at the primary care level’. So, it’s not without arguments and disputes, with fights over who should be responsible for what. –* GP *#*2.

The weakened collaboration has negative consequences for the patients, who sometimes fall through the cracks when no one wants to take responsibility for them. To an increasing extent, the GPs also feel left alone with difficult patients, without receiving the necessary support from secondary care.

*The danger with care agreements is that everyone focuses on their own part, which leads to a no man’s land. I’ve worked so long that I remember when the GP did the best he could, and when he reached the limits of his capacity, he could refer the patient. I miss that. –* GP *#*10

### 2. Guidelines promote measurable over unmeasurable knowledge

The second main theme revolves around the tension between measurable and non-measurable knowledge. The GPs provided numerous examples of how they utilize capacities in their work that cannot be standardized or properly described through guidelines, yet are seen as equally important.

#### Algorithmic thinking may suppress other knowledge forms

Some GPs expressed that clinical guidelines, medical education, and work reviews primarily focus on improving easily measurable values in healthcare. This may lead to their work becoming more mechanical. One example of how guidelines promote this form of knowledge is the increasing use of assessment scales to support evaluation during patient encounters, as recommended in the guidelines.

*Our psychologists […] may say, ‘The patient scored very high on this assessment scale–that’s worrisome.’ But for me it’s a worthless scale. A patient who is upset with their boss and fills it out in the heat of the moment will score very high. –* GP *#*6

In this process, other important aspects of medical practice become undervalued, which may hinder the development of key skills among GPs.

*If I use a metaphor, the ability to cook food worsen if you always rely on a cookbook. But the clinical eye that comes with experience–the ability to perceive a patient, to read behind the lines, to recognize what is anxiety. Use of consultation tools. What are the patient’s fears, expectations and thoughts? It’s easy to forget about that. –* GP *#*2

The ability to see the individual patient in their context and take a holistic perspective on the situation, which is characteristic for general practice, is one skill that may be compromised in the current system. Another is the ability to support behavioural change and foster patient acceptance, both of which are important for promoting overall health.

*She had tons of papers that she had written to the Social Insurance Agency. After a while, I told her that even if she is right, it might be time to bury the hatchet and move on. ‘This is not making you feel better.’ Later, she told me that was the best advice I could have given her.* – GP #11

The tacit or practical knowledge, that the GP develop over time, was also described as important. Some of the GPs described this as the art of medicine or as relying on their intuition in the clinical situation. Such skills develop with clinical experience and through building long-term relationships with patients.

*When something feels off, but you can’t quite put your finger on what it is. Then, in the end, something usually surfaces, something unexpected.* – GP nr 4

#### Guidelines may impair clinical judgement

The GPs also provided examples of how strictly adhering to guidelines can actually harm the patient if not accompanied by the GP’s critical thinking and consideration the patient’s own preferences. Such harms included unnecessary over-investigations, which can cause anxiety, physical discomfort, or even pose a risk to the patient. Another concern was that current guideline-driven care contributes to increased medicalization, which may not be beneficial to the patient’s overall quality of life. There were also examples of how strict adherence to guidelines may lead to denying patients treatments that could have been beneficial.

*An elderly woman with anaemia was referred […] and they wanted to perform a bone marrow biopsy for which she was not prepared. She tried to object, though perhaps not very forcefully. It felt like the decision had already been made. She had undergone the procedure once in the past, and found it to be a terrible experience. […] She experienced severe pain. […] Afterwards, she suffered from severe back pain for many months, and no malignancy was found. I felt it was almost like abuse.* – GP #5

#### Guidelines are ill-suited for some patient categories

Many of the GPs provided examples of patient categories they frequently encounter, where knowledge from guidelines is difficult to apply. The common denominator for these patients seemed to be that their conditions do not easily fit into well-defined disease categories, but are rather multifactorial and often involve psychosocial complicating factors. Examples included patients with chronic pain, psychiatric disorders—especially those requiring prolonged sick leave certificates—multi-morbid and elderly patients and those with diffuse symptoms or functional disorders. In these cases, the doctor must let go of the guidelines and rely on experience-based practical knowledge. Discussions and collaboration with colleagues were also highlighted as important when managing patients who defy categorization.

*When encountering patients with diffuse symptoms, I gain nothing from using guidelines.*

– GP #8

### 3. Although autonomy in relation to guidelines is highly valued, there are compelling reasons to submit

The third theme relates to the tension between, on one hand, the GPs’ conviction about the positive aspects of exercising their own clinical autonomy, and on the other hand, factors encouraging them to relinquish this autonomy. Such factors include the perception that following guidelines is a duty, as well as the expectation that adhering to them will result in a less demanding work and a reduced personal responsibility.

#### The GPs value and exercise a high degree of autonomy in clinical, patient-related work

The GPs expressed that they value and exercise a high level of autonomy in relation to guidelines when making direct patient-related decisions. This was considered vital to be able to work efficiently and make the right decisions in the individual situation, even if it directly conflicts with guidelines.

*I can be generous with interventions that are cheap, easy, and not too harmful, such as X-ray examinations, if the patient is hesitant to resume movement after an accident. […] Medically, it might not be strictly necessary, but on the other hand, it might be required for this patient to start rehabilitation. – GP #11*

Common situations in which the GPs decide not to adhere to guidelines included when the demands of the guidelines are difficult to fulfil due to time constraints or when the GP did not perceive the health outcome for the patient as worth their work and time investment. A recurring example illustrating this was the decision not to pursue treatment targets concerning cholesterol levels. When considering deviation from guidelines, the GPs emphasized their professional judgement and responsibility for the situation.

*Imagine people with asthma and diabetes. They are supposed to have a yearly check-up with both a doctor and a nurse. But it is not possible due to time constraints. […] Then it is my professional judgement to decide that this is enough, even if it means poor statistics in the quality registry. – GP #3*

The patient’s wishes also had a significant impact on their decisions, and respect for patient autonomy was often prioritised over the guidelines’ instructions. This was especially true when the patient declined an investigation or treatment, even if, in the GP’s opinion, it could be harmful to the patient.

*I’m not so insistent anymore. […] I recommend them to, but if they don’t want the cholesterol-lowering medicine, I don’t persuade them anymore. – GP #2*

GPs’ willingness to adhere to guidelines may also be affected by doubts about the guideline-making process. Some doubted whether there are always only good motives behind the guidelines, or if there could be financial motives, such as lowering the threshold for medical treatment. Others questioned whether the committees creating them always consist of people with sufficient scientific knowledge. This view was especially prominent when it came to local guidelines compared to national ones.

*I realize the danger of poor-quality guidelines, and one such danger is that people lacking sufficient scientific knowledge create them.* – GP #1

Situations in which the GPs perceived that management control restricts their freedom to make independent decisions and manage their own work often evoked negative emotions. These emotions included irritation, anger, shame, worry, and frustration.

*I can feel a growing irritation when I want to make my own decisions. If your patient has asthma, you must do this, that and that. […] It gets boring and mechanized.* – GP #8

#### Coping with high demands through adaptation

There were, however, examples where the GPs described themselves as small pieces in a puzzle, or as cogs in the machine, whose purpose it is to do the task they have been assigned in order to maintain the functionality of the healthcare system. This view included the belief that one should not make decisions that stray too far from the instructions in the guidelines. Additionally, there was a perception that, at times, following guidelines’ instructions makes work easier and more efficient in clinical situations than using critical thinking and relying on one’s own judgement.

*It might be an easy way out. Instead of addressing the underlying problem, just: ‘We order some tests’, ‘Yes they were normal’ then ‘Thank you and goodbye’.* – GP #2

Delegating decision-making to guidelines was also described as an easy way to alleviate the GP’s responsibility for the medical outcome. Similarly, referring to guidelines in discussions with patients can relieve the GP of responsibility for the decision when the patient holds an opposing view.

*Regarding opioid prescription, this clear guideline is helpful. […] If you have a chronical, non-cancerous pain: ‘No’. In that case, it is helpful to refer to the guideline. –* GP #6

#### The degree of autonomy is influenced by personal experience, fear of making mistakes and local workplace-culture

Even though the GPs highly value clinical autonomy in relation to guidelines, they provided examples of factors that can affect the autonomy. One such factor was the level of experience of the GP. Growing experience leads to increased self-esteem, which can make the GP more autonomous in the decision-making. To actively practice one’s own autonomy can also strengthen it.

*It has been an ongoing process for me to not just comply […] I have had to practice not following the guidelines to the letter. – GP #2*

Some of the GPs also described observing that other doctors with certain personal traits—namely meticulousness and anxiousness—tend to follow guidelines more slavishly.

*Such doctors have always existed, with or without guidelines – those who are very focused on following a ‘cookbook’ that tells them how to work, driven by a fear of making mistakes. But I think these guidelines are an extra trigger for them.* – GP #2

The degree of clinical autonomy exercised by the GPs also appeared to be influenced by their group of colleagues, who shape the local workplace culture. Some GPs had experienced a range of attitudes to following guidelines across collegial groups.

*If you work in a primary care centre where the focus is on the guidelines, it will be in one way. If you work in place where the focus is less pronounced, perhaps with more emphasis on continuity of care and treating patients as individuals, then it will be in another way.– GP #7.*

## Discussion

In this study, we have shown that within the current guideline-driven management control of healthcare, GPs find themselves caught in the midst of three areas of tension between conflicting values. These areas of tension— (1) Torn between high ambitions and their resulting negative side-effects, (2) Guidelines promote measurable over non-measurable knowledge, and (3) Although autonomy in relation to guidelines is highly valued, there are compelling reasons to submit—raise various challenges, which are further exacerbated as guidelines continue to grow in both number and complexity.

### Findings in relation to previous research

#### Theme 1: Torn between high ambitions and their resulting negative side-effects

Regarding the first field of tension, this study confirm finding in earlier studies that GPs generally appreciate the development of and use of evidence-based clinical guidelines, and believe that it ultimately benefits patients by improving quality in healthcare [25, 37, 38]. The negative effect in the form of increased workload resulting from the growing number and complexity of guidelines, as shown in our results, has also been acknowledged in other qualitative research [29, 30]. A recent quantitative study from England has confirmed that GPs have experienced a substantial increase in total workload per patient during the period 2005–2019. Notably, indirect workload (administrative patient-related work) per patient-year increased by 172% during this period [39]. Furthermore, quantitative analyses have shown that, given current staffing levels, it is impossible for GPs to fully comply with all guideline instructions due to time constraints [40, 41]. The increase in workload for GPs has earlier been highlighted as contributing to both deteriorated work environment and reduced patient safety, with some describing the situation as being highly problematic [22, 29, 30]. This situation has prompted a recent initiative to incorporate ‘Time Needed to Treat’ into the development of clinical guidelines [42].

Considering both current and previous studies, one type of guideline emerges as particularly double-edged: those that delineate the areas of responsibility for GPs versus secondary care physicians and define criteria for valid referrals. These care agreements, prevalent in Sweden, have been described in earlier qualitative studies as making GPs feel controlled, leading to frustration and irritation [38]. At the same time, Swedish GPs have also been shown to frequently omit required information in referrals [43]. The GPs in our study perceive these guidelines as having become increasingly detailed in recent years and identified several problems associated with them. The referral process becomes more complicated and time-consuming, but they also led to fragmented healthcare, with GPs experiencing worsening collaboration with secondary care physicians and patients falling through the cracks. Fragmented healthcare has earlier been described as both a reason for deteriorating work environment for GPs and a threat to patient safety [29, 44]. However, to our knowledge, these kinds of care agreements have not been extensively discussed in previous research, nor does there appear to be a commonly used term to refer to them.

#### Theme 2: Guidelines promote measurable over unmeasurable knowledge

The findings in our study, suggesting that guidelines may direct attention towards measurable knowledge on behalf of other knowledge and abilities important for health outcomes, have to some extent been reflected in earlier research. It has previously been highlighted that guidelines may divert the GP’s attention during consultations, constraining their flexibility and limiting opportunities for creative problem-solving [25]. Furthermore, the increasing use of clinical guidelines has been identified as a factor that may hinder the GP’s ability to exercise empathy, a quality linked to positive outcomes such as higher patient satisfaction, a deeper understanding of the patient, and improved adherence to proposed treatments [28]. Additionally, GPs have previously expressed concerns that guidelines may potentially jeopardize the doctor-patient relationship [23]. This relationship is itself believed to be important for patient health. One reason is that continuity of care—often regarded as a proxy measure for the doctor-patient relationship—has been associated with favourable outcomes, including fewer hospital admissions and lower mortality rates [44, 45]. Our study supports these findings by emphasizing unmeasurable values such as the importance of building long-term relationships with patients, but also the role of clinical judgement during consultations, the use of experience-based practical knowledge, and the need to maintain a holistic perspective.

Regarding the risk that the use of clinical guidelines may potentially harm patients by impairing clinical judgement, earlier studies have primarily identified this as a risk for overtreatment, polypharmacy, and the medicalization of patients [22]. This study adds examples of how the risk of subjugating patients to unnecessary and potentially harmful investigations increases when their preferences are not considered in medical decision-making. However, similar to this study, respect for patient autonomy over strict adherence to guidelines appears to be common in previous studies involving GPs—a tendency not always observed in studies of other medical specialists [16, 25, 27, 46].

On a more theoretical level, the discrepancy between knowledge rooted in traditional quantitative scientific research method, on the one hand, and the art of medicine—which includes tacit and practical knowledge derived from clinical experience—on the other, has been widely discussed [10, 47]. That our study highlights the tendency of clinical guidelines to favour measurable over unmeasurable knowledge reflects discussions from fields such as sociology and philosophy. It has been argued that the pursuit of measurability can influence how people think and act, potentially narrowing the appraisal of relevance and value to what is easily measured, at the expense of other ways of knowing [48, 49].

#### Theme 3: Although autonomy in relation to guidelines is highly valued, there are compelling reasons to submit

Regarding the tension between exercising and subjugating clinical autonomy in relation to guidelines, earlier research has shown concerns among GPs that their clinical autonomy is being challenged by reorganization of work, including increasing external quality controls and demands for accountability. As a result, they sometimes struggle to maintain their autonomy when clinical guidelines conflict with their own clinical judgement, and guidelines have even been proposed as introducing the policy-maker as a third decision-maker in the consultation [15, 16, 20, 25, 50]. However, the extent to which clinical autonomy has been affected remains an open question, as GPs in other research both report and are shown to frequently make exceptions to guideline instructions [15, 20, 25, 26, 37]. According to the findings of this study, the GPs generally still consider their clinical autonomy to be strong. They do not hesitate to make decisions contrary to the instructions of clinical guidelines if they believe it is the right course of action in the situation, weather based on their clinical judgement or due to distrust in the legitimacy of the guidelines. One possible explanation for this strong sense of autonomy could be that all GPs in our study were experienced specialists, in particular because they also expressed the view that junior doctors tend to find it more difficult to refrain from using guidelines.

What complicates matters, however, is that the GPs also describe situations where they voluntarily refrain from using their clinical discretion and instead uncritically follow clinical guidelines—not because they believe it is the best course of action, but because it may be the easiest or most convenient option in the situation. They also provide examples of how this may relieve them of responsibility for the decision, both in the event of an external audit and in their interaction with the patient. This way of acting could be interpreted as functional stupidity, a concept described by Alvesson and Spicer as a feature of contemporary organizations that discourages reflexivity, substantive reasoning and justification of action. While functional stupidity comes with certain benefits for the individual, such as increased sense of confidence and certainty, as well as for the organization, it can potentially create problems within both the organization as well as internal dissonance for the individual [51].

### Strengths and limitations

This study aimed to explore how guideline-driven management control affects the practice of the GP, and the qualitative design featuring semi-structured interviews enabled personal and uncensored reflections. This led to revelations of mistakes and other shortcomings that may have been missed had the interviews been conducted as focus-group interviews, a design commonly used in earlier studies on the subject [22, 23, 27]. The decision to include only specialists in general practice, combined with the interviewer being a specialist himself, ensured a deep understanding of the field being investigated and minimized the risk of misunderstandings. Another strength is the variation in the composition of the participants in terms of geographical location, age and sex, as well as the inclusion of GPs from both privately and publicly run primary care centres of varying sizes. Additionally, since the study included both GPs with experience from guideline development and those without, the likelihood of capturing a broad range of views and perspectives increased. This was confirmed by the diverse opinions expressed by the participants regarding the inherent value of strictly adhering to guidelines.

Compared to most earlier studies on the subject, this study let the participants themselves define the concept of guidelines. Earlier studies have, with a few notable exceptions, commonly focused on certain forms of guidelines, such as prescription guidelines or guidelines for single diseases [16, 22, 23, 27]. By abstaining from this approach, we increased the likelihood of not missing out important aspects of guideline-driven management control, and finding new and unexpected results. Another approach that distinguished this study from most previous ones is that we, as researchers, aimed to maintain a neutral stance toward the inherent value of different forms of clinical guidelines throughout the study. In contrast, a common approach in previous research has been to investigate barriers to the use of guidelines, thereby implicitly adopting a positive stance towards the value of guidelines [23].

Qualitative research provides in-depth understanding of a specific context rather than broad generalisations. By providing rich descriptions of the data, participants, and the context where the study is situated, the reader can assess whether the findings are transferable to their context [52]. The findings of this study convey a variety of perceptions, but may not be transferable to all GPs working in Sweden. Furthermore, since only specialists in general practice were included in this study, the findings may not reflect the experience of more junior doctors, who constitute a large proportion of those working in primary care, thereby further limiting the generalisability of the results.

Since the results are based on opinions and experiences of the participants, it is important to keep in mind that the findings—such as those regarding effects of guidelines on the healthcare system—may be influenced by the current societal debate, such as the discussion around initiatives like ‘Choosing Wisely’ [53]. There may also be a risk that participants are more inclined to focus on the negative aspects of guidelines rather than using their interview time to discuss their positive aspects. Some participants clearly recognized this risk, as they occasionally made a point of emphasizing that they fundamentally appreciate guidelines.

There is also a risk that we, as researchers, may have been influenced by our own preconceptions about the subject, particularly since half of the group are GPs and are therefore affected by guidelines in their daily practice. These preconceptions may also have been reinforced during the literature search, as many of the relevant studies examining the impact of guidelines on primary care focus on barriers to their use. During the interviews, the interviewer’s dual role as both GP and researcher may have introduced subtle bias, such as influencing the participants to provide socially desirable responses.

Finally, the credibility of the study could have been strengthened through methodological triangulation by supplementing the interview data with other sources of information, such as observations or document analyses, or by using alternative interview formats, such as focus groups. It could also have been enhanced if the interviews had been conducted by different authors, thereby broadening the perspectives represented.

## Conclusions

Our study indicates that the guideline-driven management of healthcare presents several challenges that are important for healthcare decision-makers and guideline developers to acknowledge in the pursuit of higher quality care and more effective implementation of biomedical research findings. The GPs in our study all agree that evidence-based clinical guidelines have significantly contributed to healthcare and serve as a vital resource for updated medical knowledge. However, our results also illustrate, through concrete examples, how GPs perceive that these increased ambitions can lead to adverse effects. By increasing the workload, this development contributes to a deteriorated work environment, as well as crowding-out effects. Also, the expanding use of so-called care agreements further contributes to fragmented healthcare, reducing flexibility in collaboration. Guidelines may also unintentionally lead to the underdevelopment of skills such as experience-based, contextual clinical judgement, tacit knowledge, and the relational abilities that support the doctor-patient relationship.

Between the positive and negative aspects of guidelines, the GPs attempt to strike a balance in determining how much autonomy to exercise in relation to them. While all report a high degree of clinical autonomy, many also describe situations in which, for the sake of convenience, they voluntarily suppress their clinical judgement and adhere to the guidelines. As efforts to integrate new biomedical research into practice continue to grow, the normative question of how much autonomy GPs should retain in relation to guidelines remains an issue that warrants ongoing attention.

## Supplementary Information

Supplementary Material 1.

Supplementary Material 2.

## Data Availability

While full transcripts are not publicly available due to confidentiality concerns, pseudonymised transcripts are available upon reasonable request from the Department of Public Health and Caring Sciences, Uppsala University, Uppsala, Sweden. Pseudonymised excerpts supporting the findings are included in the article.

## Abbreviations

GP: General practitioner
EBM: Evidence-based medicine

## References

[1] Blomqvist P, Winblad U. Have the welfare professions lost autonomy? A comparative study of doctors and teachers. J Soc Policy. 2024;53(1):64–85.

[2] Pickard S. The professionalization of general practitioners with a special interest: rationalization, restratification and governmentality. Sociology. 2009;43(2):250–67.

[3] Numerato D, Salvatore D, Fattore G. The impact of management on medical professionalism: a review. Sociol Health Illn. 2012;34(4):626–44. 21929618 10.1111/j.1467-9566.2011.01393.x

[4] Watt I, Nettleton S, Burrows R. The views of doctors on their working lives: a qualitative study. J R Soc Med. 2008;101(12):592–7. 19092029 10.1258/jrsm.2008.080195 PMC2625381

[5] Anell A. Vården är värd en bättre styrning. Stockholm: SNS Förlag; 2020. 144 p.

[6] Kuhlmann E, Burau V. THE ‘HEALTHCARE STATE’ IN TRANSITION: National and international contexts of changing professional governance. Eur Soc. 2008;10(4):619–33.

[7] Freidson E. Professionalism: The Third Logic. 1. Aufl. s.l.: Polity; 2013. p. 453.

[8] Forssell A, Ivarsson Westerberg A. Administrationssamhället. 1. uppl. Lund: Studentlitteratur; 2014.

[9] Power M. The audit society — second thoughts. Int J Audit. 2000;4(1):111–9.

[10] Sackett DL, Rosenberg WMC, Gray JAM, Haynes RB, Richardson WS. Evidence based medicine: what it is and what it isn’t. BMJ. 1996;312(7023):71–2. 8555924 10.1136/bmj.312.7023.71 PMC2349778

[11] Busse R, Klazinga N, Panteli D, Quentin W, editors. Improving healthcare quality in Europe: characteristics, effectiveness and implementation of different strategies. Copenhagen, Denmark: WHO Regional Office for Europe; 2019. 419 p. (Health policy series). 31721544

[12] Fernandez A, Sturmberg J, Lukersmith S, Madden R, Torkfar G, Colagiuri R, et al. Evidence-based medicine: is it a bridge too far? Health Res Policy Syst. 2015;13(1):66. 26546273 10.1186/s12961-015-0057-0 PMC4636779

[13] Kredo T, Bernhardsson S, Machingaidze S, Young T, Louw Q, Ochodo E, et al. Guide to clinical practice guidelines: the current state of play. Int J Qual Health Care. 2016;28(1):122–8. 26796486 10.1093/intqhc/mzv115 PMC4767049

[14] Pope C. Resisting evidence: the study of evidence-based medicine as a contemporary social movement. Health. 2003;7(3):267–82.

[15] Carlsen B. The last frontier? Autonomy, uncertainty and standardisation in general practice. Health Sociol Rev. 2010;19(2):260–72.

[16] Solomon J, Raynor DK, Knapp P, Atkin K. The compatibility of prescribing guidelines and the doctor-patient partnership: a primary care mixed-methods study. Br J Gen Pract. 2012;62(597):e275–81. 22520915 10.3399/bjgp12X636119 PMC3310034

[17] 1. Key definitions decision-making support tools | Right Decisions. Available from: https://rightdecisions.scot.nhs.uk/ggc-clinical-guidelines/clinical-guideline-toolkit/1-key-definitions-decision-making-support-tools/. Cited 2025 Mar 24

[18] Guerra-Farfan E, Garcia-Sanchez Y, Jornet-Gibert M, Nuñez JH, Balaguer-Castro M, Madden K. Clinical practice guidelines: the good, the bad, and the ugly. Injury. 2023;54:S26–9. 35135686 10.1016/j.injury.2022.01.047

[19] Southon G, Braithwaite J. The end of professionalism? Soc Sci Med. 1998;46(1):23–8. 9464665 10.1016/s0277-9536(97)00131-7

[20] Lewis JM, Marjoribanks T, Pirotta M. Changing professions: general practitioners’ perceptions of autonomy on the frontline. J Sociol. 2003;39(1):44–61.

[21] Checkland K. National service frameworks and UK general practitioners: street-level bureaucrats at work? Sociol Health Illn. 2004;26(7):951–75. 15610474 10.1111/j.0141-9889.2004.00424.x

[22] Austad B, Hetlevik I, Mjølstad BP, Helvik AS. Applying clinical guidelines in general practice: a qualitative study of potential complications. BMC Fam Pract. 2016;17(1):92. 27449959 10.1186/s12875-016-0490-3 PMC4957916

[23] Carlsen B, Glenton C, Pope C. Thou shalt versus thou shalt not: a meta-synthesis of GPs’ attitudes to clinical practice guidelines. Br J Gen Pract. 2007;57(545):971–8. 18252073 10.3399/096016407782604820 PMC2084137

[24] Brante T, Johnsson E, Olofsson G, Svensson L. Professionerna i kunskapssamhället: en jämförande studie av svenska professioner. Första upplagan. Stockholm: Liber; 2015;342 p.

[25] Tracy CS, Dantas GC, Upshur RE. Evidence-based medicine in primary care: qualitative study of family physicians. BMC Fam Pract. 2003;4(1):6. 12740025 10.1186/1471-2296-4-6 PMC165430

[26] Lugtenberg M, van Zegers- Schaick JM, Westert GP, Burgers JS. Why don’t physicians adhere to guideline recommendations in practice? An analysis of barriers among Dutch general practitioners. Implement Sci. 2009;4(1):54. 19674440 10.1186/1748-5908-4-54 PMC2734568

[27] Carlsen B, Norheim OF. What lies beneath it all?” – an interview study of GPs’ attitudes to the use of guidelines. BMC Health Serv Res. 2008;8(1):218. 18945360 10.1186/1472-6963-8-218 PMC2577651

[28] Derksen F, Bensing J, Kuiper S, Van Meerendonk M, Lagro-Janssen A. Empathy: what does it mean for GPs? A qualitative study. Fam Pract. 2015;32(1):94–100. 25448162 10.1093/fampra/cmu080

[29] Doran N, Fox F, Rodham K, Taylor G, Harris M. Lost to the NHS: a mixed methods study of why GPs leave practice early in England. Br J Gen Pract. 2016;66(643):e128–35. 26740606 10.3399/bjgp16X683425 PMC4723211

[30] Croxson CH, Ashdown HF, Hobbs FR. GPs’ perceptions of workload in England: a qualitative interview study. Br J Gen Pract. 2017;67(655):e138–47. 28093422 10.3399/bjgp17X688849 PMC5308120

[31] Primärvården i Europa En översikt av primärvårdens finansiering, organisation, roll och funktion i ett antal Europeiska OECD-länder. Myndigheten för vård- och omsorgsanalys; 2017.

[32] Vården ur primärvårdsläkarnas perspektiv. International Health Policy (IHP) 2022. Myndigheten för vård- och omsorgsanalys; 2023.

[33] Sverige. God och nära vård: en primärvårdsreform. Stockholm: Norstedts Juridik; 2018. p. 539.

[34] Primärvårdens resurser, styrning och organisation. Myndigheten för vård- och omsorgsanalys; 2017.

[35] Malterud K, Siersma VD, Guassora AD. Sample size in qualitative interview studies: guided by information power. Qual Health Res. 2016;26(13):1753–60. 26613970 10.1177/1049732315617444

[36] Braun V, Clarke V. Using thematic analysis in psychology. Qual Res Psychol. 2006;3(2):77–101.

[37] Luijks H, Lucassen P, Van Weel C, Loeffen M, Lagro-Janssen A, Schermer T. How GPs value guidelines applied to patients with multimorbidity: a qualitative study. BMJ Open. 2015;5(10):e007905. 26503382 10.1136/bmjopen-2015-007905 PMC4636666

[38] Ingemansson M, Bastholm-Rahmner P, Kiessling A. Practice guidelines in the context of primary care, learning and usability in the physicians’ decision-making process – a qualitative study. BMC Fam Pract. 2014;15(1):141. 25143046 10.1186/1471-2296-15-141 PMC4236535

[39] De Dumast L, Moore P, Snell KI, Marshall T. Trends in clinical workload in UK primary care 2005–2019: a retrospective cohort study. Br J Gen Pract. 2024;74(747):e659-65. 38621809 10.3399/BJGP.2023.0527 PMC11388090

[40] Porter J, Boyd C, Skandari MR, Laiteerapong N. Revisiting the time needed to provide adult primary care. J Gen Intern Med. 2023;38(1):147–55. 35776372 10.1007/s11606-022-07707-x PMC9848034

[41] Petursson H, Getz L, Sigurdsson JA, Hetlevik I. Current European guidelines for management of arterial hypertension: are they adequate for use in primary care? Modelling study based on the Norwegian HUNT 2 population. BMC Fam Pract. 2009;10(1):70. 19878542 10.1186/1471-2296-10-70 PMC2774288

[42] Johansson M, Guyatt G, Montori V. Guidelines should consider clinicians’ time needed to treat. BMJ. 2023;3:e072953. 10.1136/bmj-2022-072953 36596571

[43] Mohaddes DB Lisa Andersson, Susanne Beischer, David Sundemo, Olof Thoreson, Maziar. Brister i remisser för ortopedisk bedömning av knäledsartros. Läkartidningen. 2025. Available from: https://lakartidningen.se/klinik-och-vetenskap-1/artiklar-1/originalstudie/2025/03/brister-i-remisser-for-ortopedisk-bedomning-av-knaledsartros/. Cited 2025 Apr 28 40070301

[44] Prior A, Vestergaard CH, Vedsted P, Smith SM, Virgilsen LF, Rasmussen LA, et al. Healthcare fragmentation, multimorbidity, potentially inappropriate medication, and mortality: a Danish nationwide cohort study. BMC Med. 2023;21(1):305. 37580711 10.1186/s12916-023-03021-3 PMC10426166

[45] Pereira Gray DJ, Sidaway-Lee K, White E, Thorne A, Evans PH. Continuity of care with doctors—a matter of life and death? A systematic review of continuity of care and mortality. BMJ Open. 2018;8(6):e021161. 29959146 10.1136/bmjopen-2017-021161 PMC6042583

[46] Sanders T, Harrison S, Checkland K. Evidence-based medicine and patient choice: the case of heart failure care. J Health Serv Res Policy. 2008;13(2):103–8. 18416916 10.1258/jhsrp.2008.007130

[47] Malterud K. The art and science of clinical knowledge: evidence beyond measures and numbers. Lancet. 2001;358(9279):397–400. 11502338 10.1016/S0140-6736(01)05548-9

[48] Espeland WN, Stevens ML. A sociology of quantification. Eur J Sociol. 2008;49(3):401–36.

[49] Bornemark J. The Limits of Ratio: An Analysis of NPM in Sweden Using Nicholas of Cusa’s Understanding of Reason. In: Ajana B, editor. Metric Culture. Emerald Publishing Limited; 2018. p. 235–53. 10.1108/978-1-78743-289-520181013/full/html. Cited 2025 Apr 23

[50] Harrison S, Dowswell G. Autonomy and bureaucratic accountability in primary care: what English general practitioners say. Sociol Health Illn. 2002;24(2):208–26.

[51] Alvesson M, Spicer A. A stupidity-based theory of organizations. J Manage Stud. 2012;49(7):1194–220.

[52] Graneheim UH, Lundman B. Qualitative content analysis in nursing research: concepts, procedures and measures to achieve trustworthiness. Nurse Educ Today. 2004;24(2):105–12. 14769454 10.1016/j.nedt.2003.10.001

[53] ABIM Foundation. Choosing Wisely Initiative. Available from: https://abimfoundation.org/what-we-do/choosing-wisely. Cited 2025 Apr 8

