# Supplementary material for: General practitioners and management control through guidelines: a qualitative study of its effects on their practice
Source: BMC Prim Care. 2026 Jan 10;27:30. doi: 10.1186/s12875-025-03171-8 (PMC12857014; doi:10.1186/s12875-025-03171-8)
Supplement: Supplementary file 2 — Supplementary Material 2. [file 12875_2025_3171_MOESM2_ESM.docx]

Interview guide

**Opening questions**

- I’d like to begin by asking you to describe a patient encounter that you, for any reason, found difficult
  - What was it that made the encounter difficult?
  - How did you handle the situation, and what knowledge or clinical experience did you draw on during the encounter?
  - How did you acquire the skills or knowledge to respond in that way?
  - What challenges, if any, do you face when trying to apply that strategy?
  - Are there any conflicting interests you need to consider in such a case?
  - Can you apply clinical practice guidelines when working with a patient like this?

**Autonomy**

- Are there other parties, beyond you and the patient, that seeks to influence your clinical decision-making?
  - Who are they, and how do they influence you?
- Do you have any concrete examples from your practice where you felt pressured to act against your own conviction of what was right in the situation, or where guidelines limited your ability to help a patient in the way you deemed appropriate?
  - Can you explain why that occurred?
  - How would you have preferred to act?
- Can you describe a specific case where you made a decision that contradicted the clinical guidelines for the situation?
  - What was your reason for acting like that?
  - How did it feel to act like that?
  - How did you justify your decision, and how did you record it?
  - How would you act if faced with the same situation today?
- Can you provide any example of a patient-related decision you made that was rejected by another party?
  - How did that situation make you feel?
- Are there things you do in your daily work mainly to ensure you would pass an audit?
  - How does that impact your work?
  - What positive and negative consequences does it have?
- Can you describe a situation where you followed guidelines out of convenience, or where you limited your own autonomy because it helped relieve you of responsibility for the decision?
  - What consequences may that have?
- Could you describe an instance where following a guideline enhanced your sense of control or authority in a clinical situation?

**Management control through guidelines and medical quality**

- How do you think that the quality of care you provide is affected as guidelines become more numerous and detailed?
- In what ways is your decision-making ability impacted by an increased number of clinical guidelines?
- Can you describe an instance when adhering to a guideline led to a positive outcome, despite your initial preference to act otherwise?
- Can you describe an instance where adherence to guidelines affected a patient negatively?
- Can you think of any situation at work where you wish there were guidelines?
- Are there cases in your clinical work where you believe guidelines won’t be of any help?
- How is your indirect patient-related work affected by the increasing use of guidelines?

**Doctor-patient relationship**

- Can you describe an instance where following guidelines influenced your relationship with a patient, in a positive or negative way?

**Work environment**

- What do you enjoy about working in general practice?
- Does management control aimed at increasing adherence to guidelines affect your overall appreciation of your profession?
  - Can you give a concrete example of how it affects your work environment?
- When do you find your work as a general practitioner is meaningful?
  - What would help you feel that way more often?
- When you reflect on a patient encounter that you were satisfied with, what factors made you feel that way?
  - What factors would increase the chances of you feeling like that more regularly?
